# Updated Estimates of Radiation Risk for Cancer and Cardiovascular Disease: Implications for Cardiology Practice

**DOI:** 10.3390/jcm13072066

**Published:** 2024-04-02

**Authors:** Eugenio Picano, Eliseo Vano

**Affiliations:** 1 Cardiology Clinic, University Center Serbia, Medical School, University of Belgrade, 11000 Belgrade, Serbia; 2 Cardiology Department, Medical Faculty, Complutense University, 28040 Madrid, Spain; profvano.ucm@gmail.com

**Keywords:** atherosclerosis, cardiac imaging, cancer, radiation, risk

## Abstract

This review aims to furnish an updated assessment of the societal healthcare load, including cancer and cardiovascular disease resulting from diagnostic radiologic operations. The previously projected additional cancer risk of 0.9% in a United States 2004 study referred to radiological conditions in 1996 with an X-ray exposure of 0.50 millisievert (mSv) per capita annually. Radiological exposure (radiology + nuclear medicine) has escalated to 2.29 mSv (2016) per capita per year. Low-dose exposures were previously assumed to have a lower biological impact, since they allow the DNA repair system to mitigate molecular damage. However, epidemiological data matured and disproved this assumption, as shown by updated cancer risk assessments derived from the World Health Organization 2013 and the German Institute of Radioprotection 2014 data. The risk of cardiovascular disease aligns within the same order of magnitude as cancer risk and compounds it, as shown by a comprehensive meta-analysis of 93 studies. The collective societal burden arising from the augmented risks of cancer and cardiovascular disease attributable to diagnostic radiology and nuclear medicine is higher than previously thought.

## 1. Introduction

The cancer-attributable risk associated with diagnostic X-rays in high-income nations during the mid-1990s was approximately 2% [1]. Attributable risk is a measure indicating the disparity in cancer incidence rates between individuals exposed to a particular risk factor and those not exposed to it. For instance, if the attributable risk percentage is 5%, it signifies that 5% of cancer incidence among individuals exposed to radiation is directly attributable to that radiation exposure.

These estimates are derived from three primary sources of raw data. Firstly, estimates of the average annual frequency of exposure are considered for each type of diagnostic radiation. Secondly, assessments of organ-specific radiation doses delivered by each ionizing radiation type are taken into account. Finally, cancer incidence and all-cause mortality rates for the populations under study are analyzed.

The estimate of cancer-attributable risk is subject to increase under various circumstances. This could occur if the annual frequency of exposure rises due to heightened radiological exposure, if there are increased estimates of radiologic risk associated with any given exposure, or if both factors contribute to the rise in attributable risk. Monitoring and understanding these variables are crucial for accurately assessing and mitigating the potential risks associated with diagnostic X-ray procedures.

Notably, this risk exhibited regional variations, with rates ranging from 0.6% in the United Kingdom to 3.2% in Japan. These differences can be attributed to variations in the utilization of radiology services across regions [1]. The cardiac imaging utilization rate is skyrocketing, especially with coronary computed tomography (CT) and invasive fluoroscopy [2]. Additionally, nuclear medicine procedures now significantly contribute to total diagnostic radiation exposure [3,4]. Furthermore, risk assessments for low-dose radiation have evolved over the last two decades, with updated estimates for radiation-induced cancer [5] and a newfound awareness of previously overlooked cardiovascular disease [6]. Older estimates no longer align with current evidence. The primary aim of this paper is to offer an updated estimate of radiologic risk among cardiology patients. This estimation is grounded in emerging evidence about both cancer and atherosclerotic risks associated with low-dose radiation. Additionally, the study incorporates updated data on the volumes of radiologic exposure experienced by cardiology patients. The data sources utilized in this study encompass contemporary epidemiological evidence, recent systematic reviews, and meta-analyses, as well as recommendations from international regulatory and advisory bodies on radioprotection. Calculations regarding cancer and cardiovascular risk are derived from published comprehensive pooled analyses involving hundreds of thousands of individuals exposed to military, medical, occupational, or environmental radiation [5,6].

## 2. Historical Perspective: Fluctuations in Radiology Exposure

Over the past three decades, there has been a substantial proliferation in the number, types, and versatility of cardiac imaging techniques, many of which are often life-saving. The evolution of cardiac imaging has revolutionized the care provided to cardiology patients, albeit at the cost of increased exposure to radiation [7]. The effective radiation dose is measured in millisieverts (mSv). The mSv serves as a unit for measuring the biological effects of radiation exposure, specifically gauging the average radiation absorbed throughout the entire body. The effective dose is computed by considering doses to individual organs and their respective risk factors. This involves multiplying the dose to each organ by its corresponding risk factor. For instance, the dose to the brain is multiplied by the brain’s risk factor, and similarly for the bone marrow and other organs.

A potentially more straightforward method of conveying radiation risk involves expressing radiological doses in multiples of a chest x-ray (posteroanterior single projection). This approach has been recommended by the United Kingdom College of Radiologists and has received endorsement in the European Commission’s imaging guidelines [8]. Utilizing the “dose unit” provides a familiar metric for both medical professionals and patients. It offers a straightforward way to convey the idea that higher radiation doses correspond to an increased long-term risk of cancer [9]. This simplified communication method aids in enhancing the understanding and awareness of radiation-related risks in a comprehensible manner.

**Figure 1:**
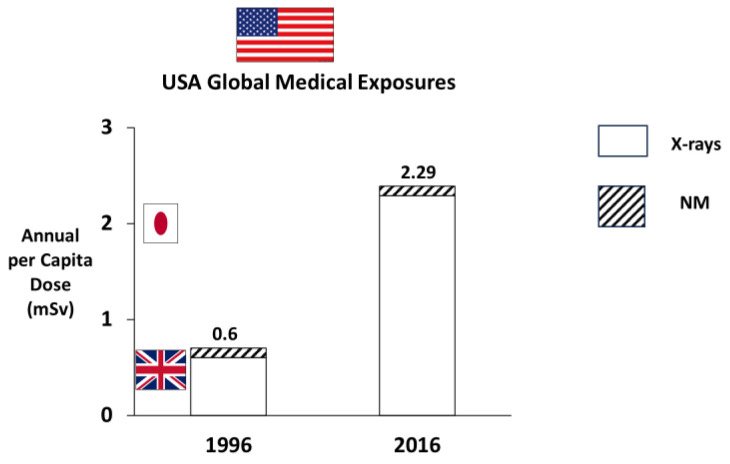
The 4-fold increase in global medical radiation exposures in the last 20 years in the USA, with marked geographical variations (lowest in the UK, highest in Japan). NM, nuclear medicine. Redrawn and adapted from original data in [1,4,7].

The United States was chosen as the reference country because of its significant role in global medical radiation procedures. The National Council on Radiation Protection and Measurements provided extensive data on radiology and nuclear medicine procedures in the United States [7]. In 1996, the estimated contribution of X-ray examinations was 0.50 mSv (equivalent to 25 chest X-rays), which subsequently increased to 3.0 mSv in 2006 [7]. Over the following decade, there was a notable transition towards more efficient scanners, heightened awareness of radiation protection within the cardiology community, and a gradual shift away from radiation-intensive tests, such as myocardial perfusion scintigraphy, to non-ionizing alternatives like stress echocardiography and cardiac magnetic resonance [7]. As a result, in 2016 in the United States, the average annual individual effective radiation dose from diagnostic radiology and nuclear medicine was 2.29 mSv, equivalent to approximately 114 chest X-rays (Figure 1). This represents a significant reduction from the 3.0 mSv (150 chest X-rays) recorded in 2006.

## 3. Reevaluation of Radiology Risk

**Figure 2:**
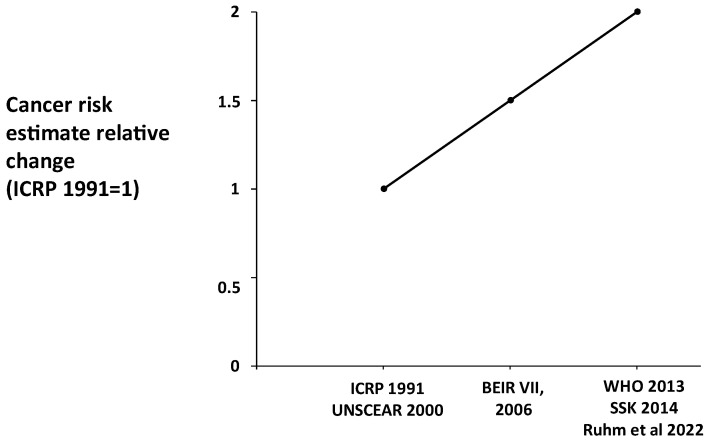
The increased estimate of radiological risk per dose observed in the last 30 years as epidemiological evidence matured. Biologic Effects of Ionizing Radiation (BEIR) report of the National Academy of Sciences [10]; ICRP, International Commission on Radiological Protection [13]; SSK, StralhenSchutzKommission, German Commission on Radiological Protection [12]; UNSCEAR, United Nations Scientific Committee on the Effects of Atomic Radiation [9].; WHO, World Health Organization [11].; Ruhm et al. [5].

The risk of cancer due to low-dose radiation exposure has doubled in the last 20 years as international bodies estimated radiation risk and set radioprotection rules [9,10,11,12,13]. For decades, a correction factor was used to reduce the risk when the available data mainly came from high-dose and high-dose-rate exposures, such as those observed in atomic bomb survivors, to account for the low-dose and low-dose-rate exposures found, for instance, in medical cohorts [11]. Initially, it was assumed that low-dose exposures had a lower biological impact, as they allow the DNA repair system to mitigate molecular damage caused by ionizing radiation [12]. However, maturing epidemiological data have refuted this assumption (Figure 2). The evidence supports the linear no-threshold model, indicating that the risk increases with the dose, even in the low-dose range.

This is underscored by Richardson et al., who conducted a pooled analysis involving over 300,000 exposed workers with individual radiation exposure monitoring data, resulting in a total follow-up of more than 10 million person-years [14]. Contrary to expectations, low-dose radiation may exert a more potent detrimental effect, possibly due to the sublethal cellular damage that is more likely to be transmitted to future cell generations than acute doses, which are more frequently lethal for the cell [14].

## 4. The Risk beyond Cancer: Cardiovascular Disease

Numerous biological mechanisms have been postulated to elucidate the association between low-dose radiation and cardiovascular disease. While the primary mechanism remains incompletely understood, various processes have been suggested, including endothelial dysfunction, inflammation, genetic instability, and an augmented occurrence of chromosome aberrations in vascular smooth muscle cells and endothelial cells’ DNA [15,16,17,18,19,20,21]. Additional proposed mechanisms encompass oxidative stress, modifications in coagulation and platelet activity, vascular senescence, apoptosis, and autophagy [22,23,24,25].

According to a model recently proposed by Andreassi, in vascular cells, low-dose ionizing radiation might initiate irreparable DNA damage and telomere erosion. This, in turn, activates a permanent deoxyribonucleic acid damage response, inducing vascular cellular senescence along with an increased senescence-associated secretory phenotype. These signals promote an intensified pro-inflammatory response within the vessel wall, thereby expediting the development of atherosclerosis and ultimately leading to cardiovascular complications [26]. While additional research is needed to refine our understanding of these intricate mechanisms, this model provides valuable insights into the potential pathways linking low-dose radiation exposure to cardiovascular disease.

In recent years, we have witnessed an alignment of the mechanisms underlying radiation-induced damage with epidemiological evidence from medical cohorts and other populations exposed to low doses and low-dose rates. A recent comprehensive meta-analysis conducted by Little and colleagues included 93 well-structured studies. For ischemic heart disease, cerebrovascular disease, and the total of cardiovascular diseases, risks increase with lower doses and lower dose rates. The risk estimates are somewhat lower but similar in scope to those for cancer mortality [27]. We have known for some time that radiation causes cancer, but today we understand that the cancer risk is higher than previously estimated. We have also known for decades that high-dose radiotherapy causes cardiovascular disease, but now we realize that even low-dose radiation accelerates cardiovascular disease.

## 5. Cumulative Cancer and Cardiovascular Risk from Radiation Exposure

The initial estimate of an additional 0.9% cancer risk associated with diagnostic radiology in the United States, as projected by Berrington de González and Darby in 2004 based on radiation exposure data from the mid-1990s [1], needs an update. The average radiation exposure from diagnostic radiology has increased significantly, from 0.50 mSv in radiology alone to 2.29 mSv from diagnostic radiology, interventional radiology, and nuclear medicine. The latest evidence suggests that, for the same dose, the cancer risk has doubled. When we calculate this, the increased risk is 4.1% with current radiation exposure and even higher at 8.2% when incorporating updated risk estimates. When considering the impact on cardiovascular disease, the combined additional risk now approaches at least 10%, accounting for both cancer and cardiovascular risks. It is important to note that we excluded data from radiotherapy (6.2 million procedures per year worldwide) and radionuclide therapy (1.4 million procedures per year worldwide) [7].

## 6. Recent, Ongoing, or Upcoming Studies

In recent years, the proliferation of radiological applications in medical practices and the heightened awareness of radiation exposure have facilitated the gathering of pertinent data concerning individuals professionally exposed to radiation, notably invasive radiologists, interventional cardiologists, electrophysiologists, and medically exposed patients, with a specific emphasis on CT and invasive fluoroscopy exposures during childhood and adolescence.

The EPI-CT study undertook the examination of a multinational cohort comprising 948,174 individuals who underwent CT scans before the age of 22 in nine European countries [28]. Radiation doses to the active bone marrow were calculated based on factors such as the scanned body part, patient characteristics, period, and CT technical parameters. The study revealed an escalating risk of hematologic malignancies in direct proportion to the number of CT examinations. Results indicate that for every 10,000 children examined today (mean dose 8 mSv), one to two individuals are anticipated to develop a hematological malignancy due to radiation exposure within the subsequent 12 years. Similar correlations were observed in the EPI-CT study for head CTs and brain tumors, showing a significant linear dose–response relationship for all brain cancers and gliomas individually [29].

Numerous observational studies have also established a direct association between radiation exposure in children with congenital heart disease and the development of cancer in radiosensitive organs receiving the highest organ dose after exposure. Notably, female breast cancer and lung cancer in both sexes exhibited heightened risks [30,31]. The HARMONIC study is actively recruiting participants from a network of eight European countries to investigate the relationship between cumulative organ dose from interventional cardiology, CT scans, and other procedures, and incident cancer. The study aims to collect data from approximately 90,000 patients who underwent cardiac fluoroscopy procedures during childhood and adolescence [32]. A subset of these patients will be examined using a molecular epidemiology approach to evaluate proximal biomarkers associated with atherosclerosis, cancer, and neurodegenerative diseases. This approach is expected to provide critical evidence regarding cancer risks following exposure to ionizing radiation and explore potential conditions predisposing individuals to cancer development [33]. Simultaneously, a study involving Italian invasive cardiologists and electrophysiologists is underway, recruiting over 500 cath lab professionals. This study evaluates early biomarkers associated with atherosclerotic, neurodegenerative, and cancer risks, facilitated by the proactive collaboration of the Italian Society of Invasive Cardiology and the Italian Society of Cardiac Electrophysiology [34,35]. These ongoing studies are poised to furnish direct evidence linking radiation exposure to adverse cancer and non-cancer health effects in cardiology patients and practitioners, thus eliminating uncertainties associated with extrapolating information from disparate fields such as nuclear power plant workers or Hiroshima bomb survivors to exposed medical doctors and patients.

## 7. How to Mitigate the Medical Radiation Burden on the Population

The collective radiation exposure of patients can be diminished by adhering to best practice guidelines, thereby mitigating low-value care, and promoting and incentivizing practices that optimize radiation doses, as mandated by the Euratom Directive 2013/59 in European countries. Initially, efforts should focus on reducing inappropriate or partially inappropriate examinations, which currently account for 10 to 50% of ionizing examinations [36,37]. These examinations not only provide low-value care, compromising patient safety, but also expose healthcare providers to potential legal ramifications.

Subsequently, wherever feasible, ionizing testing should be substituted with non-ionizing testing methods that yield comparable accuracy, such as replacing perfusion scintigraphy or computed tomography with echocardiography or cardiac magnetic resonance for assessing left ventricular function at rest or during stress [38]. Thirdly, when performing necessary ionizing tests, adherence to the optimization principle is crucial. This principle allows for a tenfold reduction in radiation dose during interventional cardiology percutaneous coronary angiography [39,40] and the adoption of near-zero fluoroscopy techniques during cardiac electrophysiology interventional procedures [41].

A fourth pivotal strategy involves technological advancements to significantly reduce radiation exposure. Photon-counting technology enables a reduction in the dose of cardiac CT [42], while the adoption of positron emission tomography (PET) instead of single-photon emission computed tomography (SPECT) achieves greater than a two-fold reduction in the dose of cardiac scintigraphy. The implementation of these strategies is essential to safeguard patient safety and align healthcare practices with legal requirements.

Mitigating the medical radiation burden constitutes one facet of a comprehensive global strategy aimed at minimizing the societal and environmental impact of cardiac imaging. For instance, in magnetic resonance imaging, gadolinium-based contrast agents have the potential to accumulate in various patient tissues including the kidney, bone, skin, lymph nodes, and potentially the brain, leading to gadolinium deposition. However, as of yet, there is no definitive evidence linking gadolinium deposition to clinical disease [43].

## 8. Implications for Practice and Policy

**Figure 3:**
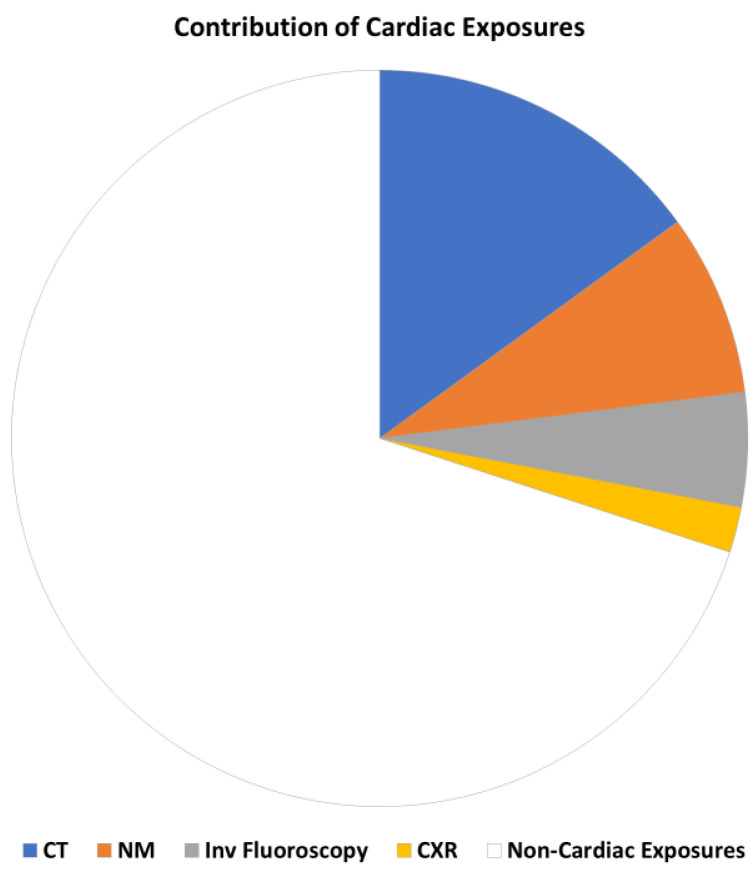
The significant contribution of cardiac exposures to overall medical exposure. Redrawn and adapted from original data in [7,44].

These findings have significant implications for routine cardiology practices. As of 2006 estimates, cardiologists account for 50% of all medical radiation exposures [44]. According to the latest available data from 2016 in the United States, cardiac nuclear medicine assessments contribute to nearly 8% of overall medical radiation exposure. This is in contrast to 15% for cardiac computed tomography, 6% for cardiac invasive fluoroscopy, and 2% for chest X-rays [7] (Figure 3).

**Figure 4:**
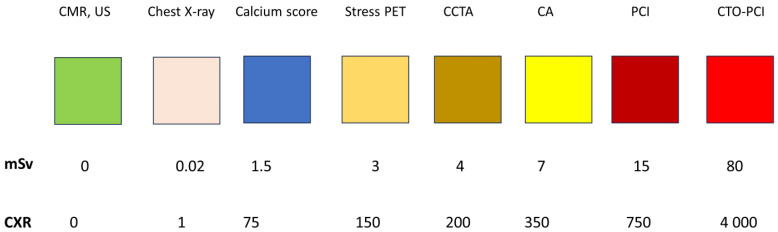
The dose associated with main cardiology tests or techniques. CA, invasive coronary angiography; CMR, cardiovascular magnetic resonance; CCTA, coronary computed tomography angiography; CXR, chest X-ray (standard postero-anterior view); CTO, chronic total occlusion of coronary artery; PCI, percutaneous coronary interventions; PET, positron emission tomography; US, ultrasound. Redrawn and adapted from original data in [7,44,45].

Some cardiac tests are associated with substantial radiation exposure, especially nuclear cardiology tests and invasive fluoroscopy techniques (Figure 4).

Notably, cardiac exposure can be substantially higher for patients in cardiology wards, occasionally exceeding 100 mSv in a single admission due to procedures such as the dilation of coronary chronic total occlusion and valvuloplasty [44]. With the advancements in cardiovascular prevention, diagnosis, and therapy, it is essential to tailor diagnostic or therapeutic interventions in cardiology to reduce the risk of cancer and the exacerbation of underlying cardiovascular conditions due to avoidable radiation exposure [46]. This is especially true for low-risk populations that make up the majority of cardiovascular testing in modern cardiac imaging practice. In the interventional era of cardiology, there was a progressive shift from cardiac to cancer deaths in our cardiology patients [45]. The trajectory toward radiological sustainability seeks to enhance patient care while minimizing the enduring risks of cancer and cardiovascular disease associated with radiation exposure [47]. This commitment ensures the holistic well-being of individuals and promotes their long-term health.

## Data Availability

Not applicable (no new data).
